# Commentary: “Neural signatures of intransitive preferences”

**DOI:** 10.3389/fnhum.2015.00509

**Published:** 2015-09-17

**Authors:** Nicholas Brown, Clintin P. Davis-Stober, Michel Regenwetter

**Affiliations:** ^1^Department of Psychological Sciences, University of MissouriColumbia, MO, USA; ^2^Department of Psychology, University of Illinois at Urbana-ChampaignChampaign, IL, USA

**Keywords:** intransitivity, decision making, irrational, preferences, neuroeconomics, risk, uncertainty

Kalenscher et al. (2010) explored the neural signatures of intransitive preferences. This endeavor is of great interest because transitivity of preferences has long been considered a key feature of rationality. The success of this approach hinges upon appropriate methods for identifying decision makers with intransitive preferences. The authors invented a descriptive numerical “index of intransitivity” (henceforth labeled K. Index) that we argue is not grounded in a quantitative model of choice behavior. We revisit the authors' original data and reclassify the participants using two well-known and well-established quantitative criteria for transitive preferences, the *random preference model* of transitive linear orders (Block and Marschak, 1960; Becker et al., 1963) and *weak stochastic transitivity* (Block and Marschak, 1960; Tversky, 1969; Iverson and Falmagne, 1985). These two models of transitive preferences embody two different ways to capture uncertainty/variability of behavior formally. According to the random preference model, preferences are probabilistic, while responses are deterministic (error-free). According to weak stochastic transitivity, preferences are deterministic and responses are probabilistic (noisy). Suitable statistical tests for these models have only recently become broadly available.

Similar to the seminal study of Tversky (1969), Kalenscher et al.'s task consisted of two-alternative forced choices among binary lotteries, with reward size and probability of a reward trading off against each other. Using a set of five lotteries, the data of interest consist of 200 responses per person, 20 repetitions for each of the 10 pairs of lotteries. Eighteen of their 30 participants scored a K. Index > 0.3 and were classified as intransitive. The authors motivated the K. Index as a means to evaluate weak stochastic transitivity.

Economists and psychologists have studied the conceptual challenges of taking a deterministic axiom like transitivity and expanding it into a probability model to incorporate the inherent uncertainty in human behavior (Luce, 1959; Block and Marschak, 1960; Luce and Narens, 1994; Loomes and Sugden, 1995; Hey, 2005). Equally importantly, recent work has provided the statistical tools needed to evaluate such models (Myung et al., 2005; Davis-Stober, 2009; Cavagnaro and Davis-Stober, 2014; Regenwetter et al., 2014). Regenwetter et al. (2010, 2011a) gave an in-depth critique of the literature on testing transitivity of preferences. These papers contributed three main points relevant to this discussion:

1. They promoted random preference models as a compelling probabilistic specification of transitivity.
2. They offered the first proper statistical test of the random preference model using “order-constrained inference” methods, and one of the first proper direct statistical tests of weak stochastic transitivity.
3. They discussed conceptual, mathematical, and statistical problems of commonly used descriptive indices similar to K. Index (see, e.g., Regenwetter et al., 2011a, Figure 4).

According to the *random preference model of transitivity* (RPT), a decision maker may have any transitive preference ≻ with (unknown) probability *P*_≻_. The binary choice probability, *p*(*x, y*) that a decision maker chooses *x* when offered the choice between *x* and *y* is the total probability of all those transitive preference states ≻ according to which *x* is preferable to *y*, i.e., *x* ≻ *y*. Formally,

(1) $$p(x,y)=\sum_{\begin{array}{c} \text{preference states}≻\\ \text{ in which }x≻y \end{array}}P_{≻}.$$

According to this model, a decision maker has probabilistic transitive preference states and responds in an error-free fashion. Understanding the mathematical and statistical properties of this model has been the subject of a sophisticated technical literature.

According to *weak stochastic transitivity* (WST), a decision maker has a single (unknown) deterministic transitive preference state. Regardless of that preference state, the decision maker gives probabilistic responses due to “errors” and the probability of an error is bounded above by $\frac{1}{2}$. Formally, for any triple of distinct alternatives, *x*, *y*, *z*,

$$\text{if }p(x,y)\geq \frac{1}{2}\text{ and }p(y,z)\geq \frac{1}{2},\text{ then }p(x,z)\geq \frac{1}{2}.$$

According to this model, a decision maker has one deterministic transitive preference state and responds in a noisy fashion. Despite appearances, this model is mathematically nontrivial. For five choice alternatives, it forms the disjoint union of 120 different hypercubes in a 10-dimensional parameter space (Regenwetter et al., 2010).

We also consider two models that permit, but do not require, intransitive preference states. Respondents with *lexicographic semiorder* preferences examine attributes (such as probability or payoff) sequentially until values on one attribute differ sufficiently (Tversky, 1969). Put very simply, a *random preference model of lexicographic semiorders* (RPLS) essentially uses lexicographic semiorders for ≻ in Equation 1 (Regenwetter et al., 2011b; Davis-Stober, 2012). A *lexicographic semiorder error model* (LSE) assumes a single (unknown) deterministic lexicographic semiorder ≻ and error-prone responses such that, essentially, *x* ≻ *y* implies $p\left(x,y\right)\geq \frac{1}{2}$. The online supplement specifies these two models fully.

Table 1 shows our reanalysis of Kalenscher et al.'s data. Columns 3–6 give each respondent's Bayes factor for each model compared to an *unconstrained model*. The latter does not constrain binary choice probabilities in any way. Following standard practice (Jeffreys, 1961), a Bayes factor larger than $\sqrt{10}$ (≈ 3.16) is considered strong evidence in favor of a given model over another. The column “Best model” provides the model with the highest Bayes factor among models supported by strong evidence. Following Cavagnaro and Davis-Stober (2014), we computed these Bayes factors using the order-constrained methodology of Klugkist and Hoijtink (2007).

**Table 1:** **Reanalysis of Kalenscher et al.'s data**.

**Participant**	**K. Index**	**RPT**	**WST**	**RPLS**	**LSE**	**Best model**
1	0.21	19.41	8.15	3.10	*0.17*	RP Transitive
2	**0.36**	*0.0027*	*0.019*	*0.01*	*0.05*	Unconstrained
3	**0.605**	<*0.001*	<*0.001*	9.01	*0.04*	RP Lexicographic semiorder
4	0.065	0.82	8.52	*0.21*	*0.083*	Weak stochastic transitivity
5	**0.30**	<*0.001*	<*0.001*	0.89	*0.08*	Unconstrained
6	**0.36**	2.21	0.92	<*0.001*	*0.01*	-
7	**0.31**	*0.019*	*0.16*	9.01	*0.24*	RP Lexicographic semiorder
8	0.005	3.57	8.53	2.49	*0.08*	Weak stochastic transitivity
9	0.26	14.77	2.44	56.28	0.40	RP lexicographic semiorder
10	**0.41**	2.11	*0.11*	<*0.001*	*0.01*	-
11	0.26	1.90	3.97	<*0.001*	*0.05*	Weak stochastic transitivity
12	0.15	5.49	8.37	3.35	*0.09*	Weak stochastic transitivity
13	**0.43**	<*0.001*	*0.002*	2.33	*0.08*	-
14	0.050	1.31	8.53	*0.18*	*0.08*	Weak stochastic transitivity
15	**0.33**	<*0.001*	<*0.001*	1.07	*0.08*	Unconstrained
16	**0.37**	<*0.001*	<*0.001*	0.66	*0.07*	-
17	**0.42**	1.67	0.42	<*0.001*	*0.01*	-
18	0.050	7.76	8.53	1.01	*0.08*	Weak stochastic transitivity
19	**0.39**	2.62	0.78	<*0.001*	*0.07*	-
20	**0.39**	1.26	0.32	*0.08*	*0.04*	-
21	**0.43**	1.19	0.45	<*0.001*	*0.01*	-
22	**0.36**	<*0.001*	<*0.001*	33.32	*0.07*	RP lexicographic semiorder
23	0.20	*0.01*	3.46	<*0.001*	*0.07*	Weak stochastic transitivity
24	**0.42**	11.21	1.44	*0.01*	*0.07*	RP transitive
25	**0.40**	3.32	*0.30*	*0.09*	*0.13*	RP transitive
26	0.29	19.54	6.84	*0.03*	*0.09*	RP transitive
27	**0.35**	6.73	1.84	*0.01*	*0.01*	RP transitive
28	**0.33**	1.30	0.48	*0.03*	*0.06*	-
29	0.25	0.35	5.29	<*0.001*	*0.03*	Weak stochastic transitivity
30	0.0067	13.65	8.53	8.91	*0.09*	RP transitive

*For each of 30 participants, we report the Kalenscher et al. degree of intransitivity score (K. Index) and Bayes factors for RPT, WST, RPLS, and LSE. We boldface K. Index values (>0.3) that fail to support transitivity. We italicize Bayes factors ($<\sqrt{0.1}$) that fail to strongly support a given model. The “Best model” column gives the model with the highest Bayes factor. When the unconstrained model is strongly supported over all four models, “Best model” gives “unconstrained.” All other cases are marked with “-” to indicate that they are not conclusive. “ < 0.001” indicates that the calculated Bayes factor is less than 0.001 indicating considerable support for the unconstrained model*.

Seven participants were classified according to a lexicographic model or the unconstrained model (i.e., allow intransitivity), compared to the K. Index which favored intransitivity for 18 participants. We selected RPT for six and WST for eight participants. The remaining nine cases produced insufficient evidence for classification. All participants we classified as unconstrained were also classified as intransitive by the K. Index. Notice the nonmonotonic relationship between the K. Index and the Bayes factors. Compare Participants 5 and 24. Participant 5 barely made it to be classified as intransitive by the K. Index while the Bayesian analysis found very strong evidence against both RPT and WST. Participant 24 had a much larger K. Index, while the Bayesian analysis strongly favored RPT and slightly favored WST. A frequentist test of each model, where applicable, yielded good agreement with the Bayesian approach (see Supplementary Table).

We recommend three refinements to the approach of Kalenscher et al. (2010). First, disregard the K. Index as a measure of intransitivity. Second, focus the fMRI analyses on only those participants for whom we identified a best model in Table 1. This recommendation might require additional participants to achieve sufficient statistical power. Third, look also for distinct neural signatures of random preference vs. error models as these models correspond to differences in the locus of choice variability: generated either via shifting preference states or error-prone responses.

## Author contributions

The first author communicated with Dr. Kalenscher, carried out the data analyses, and wrote the first draft of the paper as part of the requirements for a Masters in Statistics at The University of Missouri. The second and third authors assisted with conceptual, mathematical, and statistical approaches and contributed to the writing.

## Funding

This work was supported by National Science Foundation grants (SES-1062045, SES-1459699, PI: Regenwetter) and (SES-1459866, PI: Davis-Stober).

### Conflict of interest statement

The authors declare that the research was conducted in the absence of any commercial or financial relationships that could be construed as a potential conflict of interest.
