# Supplementary material for: Commentary: “Neural signatures of intransitive preferences”
Source: Front Hum Neurosci. 2015 Sep 17;9:509. doi: 10.3389/fnhum.2015.00509 (PMC4584983; doi:10.3389/fnhum.2015.00509)
Supplement: Supplementary file 1 [file Table1.PDF]

Online Supplement to "From descriptive indices of intransitivity to quantitative assessments: A commentary on Kalenscher et al. 2010."

Nicholas Brown, Clintin P. Davis-Stober, Michel Regenwetter

Random preference model of lexicographic semiorders (RPLS):

Let $\mathcal{LSO}$ be the (finite) collection of lexicographic semiorders, let $P_{\succ}$ denote the probability of $\succ \in \mathcal{LSO}$. The binary choice probability, $p(x, y)$ that a decision maker chooses x when offered the choice between x and y is

$$p(x,y) = \sum_{\substack{\text{preference states } \succ \in \mathcal{LSO} \\ \text{in which } x \succ y}} P_{\succ} \quad + \quad \frac{1}{2} \sum_{\substack{\text{preference states } \succ \in \mathcal{LSO} \\ \text{in which } x \not\succ y \text{ and } y \not\succ x}} P_{\succ}. \qquad (1)$$

Lexicographic semiorder error model (LSE):

Let $\succ \in \mathcal{LSO}$ be the (unknown) fixed lexicographic semiorder. The binary choice probability, $p(x, y)$ that a decision maker chooses x when offered the choice between x and y is

$$p(x,y) \begin{cases} \in [\frac{1}{2}, 1] & \text{if} \quad x \succ y, \\ \in [\frac{1}{4}, \frac{3}{4}] & \text{if} \quad x \not\succ y \text{ and } y \not\succ x, \\ \in [0, \frac{1}{2}] & \text{if} \quad y \succ x. \end{cases} \qquad (2)$$

The LSE model forms a complicated non-disjoint union of convex polytopes. While computing its Bayes factor is not computationally expensive, computing a frequentist p-value would require very extensive computation, hence we omit it.

Table 1 provides the frequentist p-values for the random preference model of transitivity (RPT), weak stochastic transitivity (WST), and the random preference model of lexicographic semiorders (RPLS).

Table 1: Reanalysis of Kalenscher et al.'s data. For each of 30 participants, we report the Kalenscher et al. degree of intransitivity score (K. Index) and p-values for goodness-of-fit tests of RPT, WST and RPLS. We boldface K. Index values (> 0.3) that fail to support transitivity. We italicize p-values less than .05, indicating lack of fit for that model. Entries with a "✓" correspond to choice data that perfectly satisfies the constraints of that model, hence a perfect fit.

Participant	K. Index	RPT	WST	RPLS
1	.21	✓	✓	.41
2	**.36**	*<.01*	*.03*	*<.01*
3	**.605**	*<.01*	*<.01*	.43
4	.065	0.12	✓	.22
5	**.30**	*<.01*	*<.01*	.05
6	**.36**	.81	.48	*<.01*
7	**.31**	*.02*	.45	*.03*
8	.005	✓	✓	.99
9	.26	✓	.56	.62
10	**.41**	.27	.20	*<.01*
11	.26	.22	✓	*.01*
12	.15	✓	✓	.32
13	**.43**	*<.01*	*<0.01*	.05
14	.050	.29	✓	.39
15	**.33**	*<.01*	*<.01*	.05
16	**.37**	*<.01*	*<.01*	*.02*
17	**.42**	.55	.72	*<.01*
18	.050	.95	✓	.69
19	**.39**	.30	.44	*<.01*
20	**.39**	.22	.36	*.03*
21	**.43**	.46	.48	*<.01*
22	**.36**	*<.01*	*<.01*	.99
23	.20	*<.01*	.66	*<.01*
24	**.42**	✓	.69	*.02*
25	**.40**	.46	.05	.17
26	.29	✓	✓	*.03*
27	**.35**	✓	✓	*<.01*
28	**.33**	.73	.44	*.04*
29	.25	.07	✓	*<.01*
30	.0067	✓	✓	.69
